# Developing Meso and Microholes by Spark-Erosion Based Drilling Processes: A Critical Review

**DOI:** 10.3390/mi13060885

**Published:** 2022-05-31

**Authors:** Sujeet Kumar Chaubey, Kapil Gupta

**Affiliations:** Department of Mechanical and Industrial Engineering Technology, Doornfontein Campus, University of Johannesburg, Johannesburg 2028, South Africa; schaubey@uj.ac.za

**Keywords:** electrode wear, material removal rate, microhole, spark erosion drilling

## Abstract

The increased demand for miniature components has drawn the attention of researchers, engineers, and industry users to manufacture precision micro and mesoholes on foils, sheets, and plates made from a variety of engineering materials. These days, micro-drilling is extensively being adopted as a fundamental operation in all kinds of smart manufacturing industries to make different types of microholes, such as through holes, blind holes, and taper holes on micro-parts and components. Drilled holes with a diameter of less than 1 mm are referred to as microholes, while drilled holes whose diameter ranges between 1 and 10 mm are known as mesoholes. Meso and microholes are commonly referred to as fine-holes. Modern or advanced drilling processes are mostly used to drill microholes from a variety of materials. This paper presents an extensive review of the previous research conducted on the drilling of fine holes (meso and micro size) by spark- erosion-based processes along with highlighting work and tool electrode materials, specifications of drilled holes, types of microholes (through or blind holes), process parameters, performance measures, and key findings. The paper aims to facilitate researchers and scholars by highlighting the capabilities of spark erosion machining, drilling, and its variants to fabricate miniature holes. The paper ends with a conclusion and future research directions to encourage further work in this area.

## 1. Introduction

Nowadays, the trend of miniaturized devices and products that are lightweight, smaller in volume, portable, cheaper, and stable in performance has become one of the fundamental necessities of several manufacturing industries, such as aerospace, automotive, medicine, biomedical, electronics, telecommunication, fluidics and consumer products [1,2,3]. Miniature deceives and products used in the abovementioned industries are printed circuit boards, pressure sensors, nozzles, accelerometers, microdies, molds, microreactors, microdroplet injectors, tooth grafting, medical appliances, fluid pumps, fuel filters, fuel injectors, optical scanners, and probes. Drilling is the most commonly used and basic machining operation in the manufacturing industries for making different types and sizes of holes on a variety of materials [4]. Drilled holes with a diameter of less than 1 mm (i.e., 1000 µm) are known as microholes [5]. Whereas, drilled holes with diameters ranging between 1–10 mm are stated as mesoholes. For the past two decades, the constraint of traditional drilling processes has become a major concern due to the requirement for higher drilling accuracy in modern industries [6]. The constraint of traditional processes for making high-quality small-sized holes or fine-holes has drawn the attention of researchers, engineers, and industrial users to explore alternative drilling processes to fabricate net-size or near-net-size microholes. The advanced micro-drilling process can produce microholes with diameters in a range of a few microns to hundreds of microns. 

### 1.1. Selection of Materials for Drilling Deep Fine-Holes

The choice and range of materials for fine holes (i.e., meso and microholes) generally depend on types of application, material features (i.e., hardness and tenacity) of workpiece and drilling tools, drilling methods, and materials cost [7,8]. Commonly, miniature-sized holes are drilled on several metallic, non-metallic, and composite metals. Metallic materials are further divided into two categories, namely ferrous and nonferrous. Ferrous metals have higher strength than non-ferrous metals and are most suitable for various construction, structural, and industrial applications [9,10,11]. These materials comprise stainless steel, high-carbon steel, high-speed steel, and cast iron. Nonferrous materials, such as copper, aluminum, lead, zinc, brass, nickel, titanium, and their alloys, are more malleable, conductive, and easily handled in diverse manners. These materials are most preferable for applications where a humid environment would oxidize ferrous metals. These materials are also suitable for electrical components due to their non-magnetization characteristics. Non-metallic materials are different types of plastics, such as Delrin, polyvinyl chloride, acrylic, fiberglass, nylon, etc. These plastics are extensively used for lightweight and noiseless applications. A composite is a mixture of two materials having different characteristics (i.e., physical and chemical). Composite materials have lightweight qualities, high strength, stiffness, and they are resistant to electricity. Metal-matrix-composite, glass fiber reinforced-composite, fiberglass, carbon-fiber-reinforced-polymer, etc. are some commonly used composite materials that are widely used in aerospace, automotives, corrosive environments, maritime environments, and electrical parts and components.

### 1.2. Procedure of Experiments and Methods of Drilling Precision Deep Fine-Holes 

The summary of the various methods for drilling miniature deep fine holes is presented in Table 1. These methods can be classified as (i) conventional drilling methods (CDMs); and (ii) advanced drilling methods (ADMs). Micro-lathe, micro-milling, and micro-drilling are the most commonly used conventional methods to produce miniature-sized holes in metals and non-metals with the help of the various drill bits. CDMs have some inherent limitations, such as tool wear, burr generations, geometrical inaccuracy, and low material removal rate. Therefore, advanced drilling methods are employed to overcome the restrictions of CDMs. ADMs can be classified as (i) mechanical erosion drilling (MED); (ii) thermoelectric erosion drilling; (iii) chemical form of drilling; and (iv) electrochemical drilling [12,13,14].

Mechanical erosion-based drillings are abrasive water jet drilling (AWJD) and ultrasonic drilling (USD). In these processes, materials are removed by the mechanical erosion of work material by high-pressure slurry (abrasive particles mixed with water). AWJD can produce miniature-sized holes on metallic and non-metallic materials. USD is used for machining miniature-sized holes on conductive and non-conductive materials having a hardness of more than 40 HRC. In thermoelectric erosion drilling methods, thermal energy is utilized to remove materials by melting and vaporization. These methods include ram-based spark-erosion machining (SEM), spark-erosion drilling (SED), and laser drilling. SEM and SED are used to produce miniature-sized holes on electrically conductive materials irrespective of their hardness, while laser drilling is used to produce miniature-sized holes on non-reflective metallic materials. The material removal rate in laser drilling is higher than in SED and SEM processes. The micro versions of SEM and SED are micro-spark-erosion machining (µ-SEM) and micro-spark-erosion drilling (µ-SED). Electrochemical drilling (ECD) is used to produce microholes on workpieces by ion displacement. It involves anodic dissolution where an electrolytic cell is formed by a tool (cathode) and workpiece (anode) surrounded by the continuous flowing electrolyte. In chemical drilling (CHD) methods, miniature-sized holes are produced by the chemical action of the corrosive agents.

**Figure 1:**
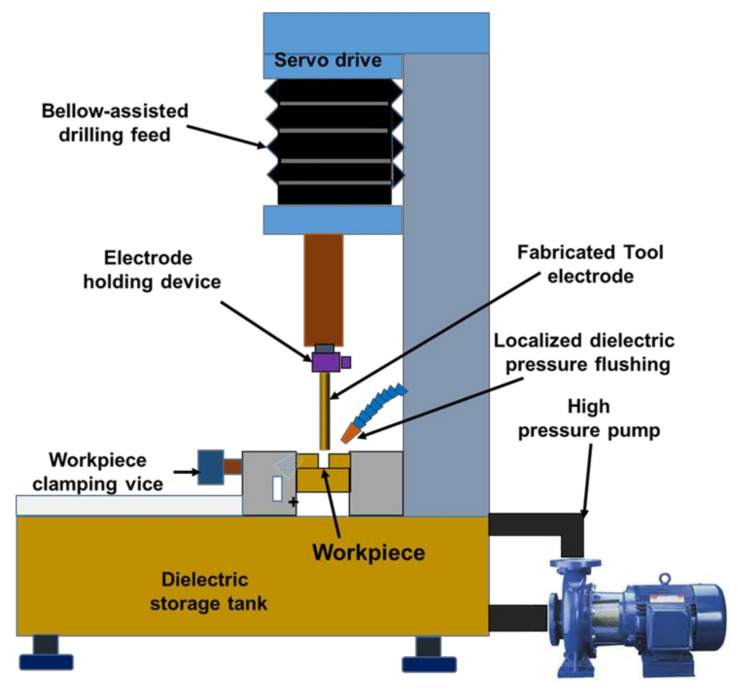
Schematic representation of micro-spark-erosion machining (µ-SEM) setup for drilling.

**Figure 2:**
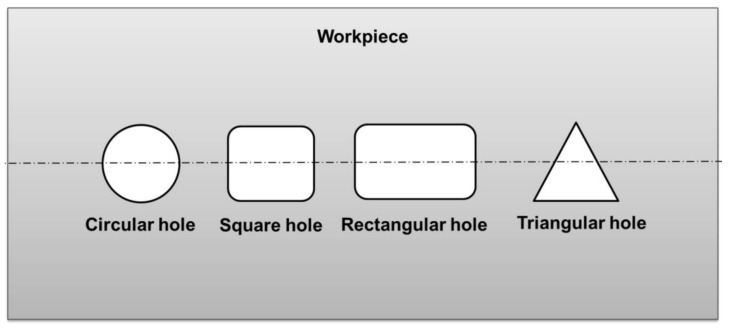
Types of holes that can be fabricated by the micro-spark-erosion machining (µ-SEM) process.

Micro spark erosion machining (µ-SEM) is used to make a different type of microhole by using similar shapes and sizes of fabricated tool electrodes. In the µ-SEM process, the non-rotating tool electrode moves downward to make a microhole on the workpiece. Figure 1 shows the schematic diagram of the µ-SEM setup. µ-SEM has the potential to fabricate circular and non-circular through and blind microholes, such as square, rectangular, and triangular as shown in Figure 2.

**Figure 3:**
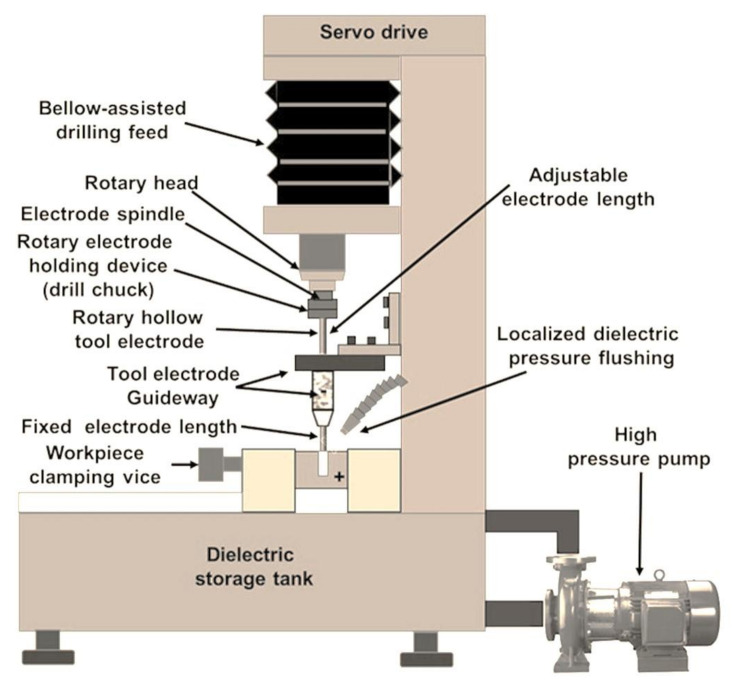
Schematic representation of micro-spark-erosion drilling (µ-SED) setup.

Micro spark erosion machining (µ-SEM) for meso and microhole drilling using hollow rotating tool electrodes is popularly known as micro spark erosion drilling (µ-SED). Figure 3 presents the schematic diagram of a µ-SED setup. Usually, µ-SED is adopted to drill fine holes (i.e., meso and micro-sized holes) in difficult-to-machine materials that are not possible to drill by traditional drilling methods. µ-SED is a non-traditional drilling method that fabricates deep and tiny holes. µ-SED can drill high aspect ratio circular and of complex geometry fine holes. µ-SED is a method of drilling fine holes on difficult-to-machine materials by recurring electric pulsed discharges or sparks, which are generated between the workpiece and rotary hollow electrode that eventually melt and vaporize the workpiece for hole making [15,16]. The basic components are (i) multi-axes worktable; (ii) rotary spindle drill chuck or collet system for holding electrodes; (iii) electrode guides made of ceramic or diamond; (iv) DC power supply; (v) capacitor box; and (vi) dielectric circulation system (flow pump). Generally, precision circular hollow bars or tubular electrodes of brass, copper, and its alloys are used as tools to remove the materials from the workpiece during drilling fine holes. These electrodes have a single hole/passage or array of passages. Hollow electrodes are used for localized flushing and cooling purposes. The array of tubes is preferred to eliminate the formation of slugs inside the passage. The tool electrode is continuously rotated during the drilling of fine holes, which enables the removal of the eroded particles from the inter-electrodes gap (IEG) and accelerates the drilling process. The ceramic or diamond guide positions the electrode tip on the exact location of the workpiece to avoid drifting during the drilling of fine holes. It also eliminates the wobbling of the rotary electrode and thus minimizes the drilling of oversized fine holes. Deionized water or hydrocarbon oil (kerosene oil) is used as dielectric to flush away the eroded particles (also referred to as debris) during the drilling process. Usually, a DC power supply is used, in which positive (anode) and negative (cathode) poles are connected to the workpiece and electrode, respectively. The process parameters are pulse-on-time, pulse-off-time, peak current, gap voltage, capacitance, the rotational speed of the tool electrode, and dielectric flow rate. These parameters can be varied during drilling fine holes. The electrode materials, dielectric fluids, workpiece thickness, and aspect ratio are other parameters, but these parameters cannot be varied during the drilling operation.

### 1.3. µ-SED Process and Working Principle

µ-SED process is similar to µ-SEM except hollow tool electrodes rotate continuously during the drilling process. In the µ-SED process, a vertical precision hollow electrode is firmly mounted into the tool chuck and attached to the spindle located along *Z*-axis. The spindle is attached with a direct current (DC) motor. The ceramic or diamond guide is used to position the electrode on the desired location of the workpiece for drilling the fine hole. The guide is located above the workpiece. The downward feed of the electrode is provided with the help of a servo drive that maintains a certain gap between the electrode and workpiece, in which sparks are generated and burn the materials from the workpiece to produce through or blind fine-hole. During drilling, the dielectric fluid continuously circulates through the rotary hollow electrode to flush away the eroded particle or debris from IEG and dissipates the heat produced by electric sparks. Only localized flushing is used in the µ-SED process. The drilling is completed after the desired depth of penetration on the workpiece by the rotary electrode and the electrode is retracted above the workpiece. The working principle of µ-SED is similar to the spark-erosion machining process (SEM) and its micro version (µ-SEM). The mechanism of material removal is based on thermoelectric erosion. A very strong electric field is formed at minimum IEG after supplying DC power to the workpiece and rotary electrode. The suspended microscopic particles in a dielectric fluid are accumulated around the location of the strongest electric field and form a conductive bridge across the electrodes gap. When the supply voltage is exceeded to dielectric breakdown voltage then the conductive bridge breaks due to excessive heat and temperature. Due to the collapse of the conductive bridge, sparks are generated between the interelectrode-gap. The material is removed from the workpiece due to melting and vaporization, and at the same time dielectric is flushed away from the eroded particles from the interelectrode-gap to ensure smooth drilling. The generation of sparks is continuously repeated until the drilling is up to the desired depth [17].

**Figure 4:**
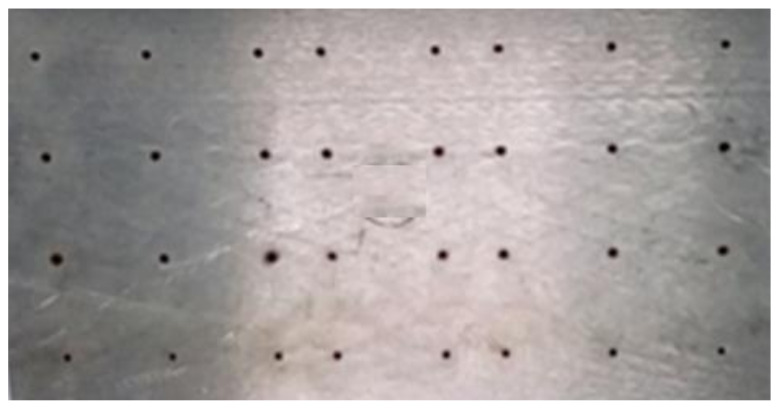
An array of microholes fabricated on SS 304 by micro spark-erosion drilling (µ-SED) process using a rotating brass hollow tool electrode of 800 µm diameter.

**Figure 5:**
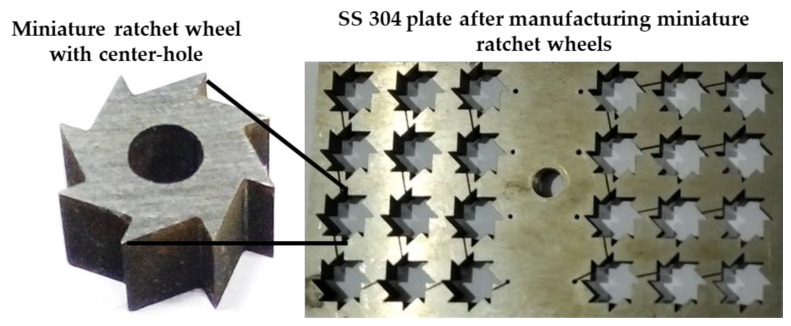
Miniature ratchet wheels manufactured by WSEM process from micro spark-erosion drilled SS 304 plate.

**Figure 6:**
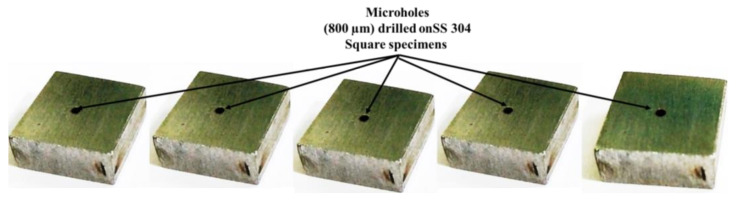
Microholes fabricated on square SS 304 specimens by micro spark-erosion drilling (µ-SED) process using rotating brass hollow tool electrode of 800 µm diameter.

**Figure 7:**
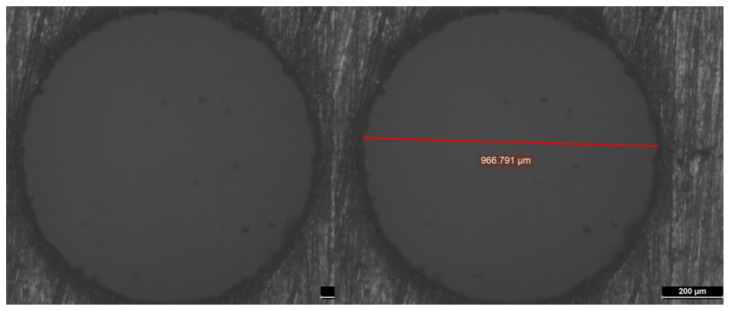
Optical images of microholes fabricated by micro spark-erosion drilling (µ-SED) process using rotating brass hollow tool electrode of 800 µm diameter.

**Figure 8:**
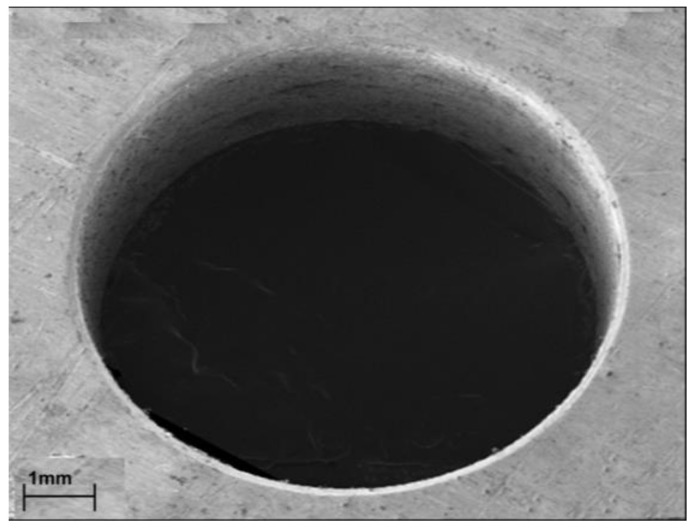
SEM images of mesohole fabricated by spark-erosion drilling (SED) process.

Figure 4, Figure 5, Figure 6, Figure 7 and Figure 8 depict some results of the completed and ongoing research work on the manufacturing of microholes on the rectangular SS 304 plate and square SS 304 specimens by µ-SED at the University of Johannesburg. Figure 4 shows the array of microholes of 800 µm diameter fabricated on a 5 mm thick stainless steel (SS 304) plate by the µ-SED process, using a rotating hollow brass electrode of 800 µm diameter. These microholes were used for wire passages to manufacture the different types of miniature gears, such as spur gears, helical gears, bevel gears, spiral gears, and ratchet wheels with center-hole by wire spark erosion machining (WSEM) process [9,10,18,19,20]. The authors manufactured all these gears by WSEM from SS 304 plate. Figure 5 shows the WSEM manufactured miniature ratchet wheel and rectangular SS 304 plate after manufacturing. Authors have conducted an experimental study to fabricate the microhole at the center of a 5 mm thick and 5 mm square SS 304 specimen by the µ-SED process, using a rotating hollow brass electrode of 800 µm diameter as shown in Figure 6. Figure 7 depicts an optical image of microholes fabricated on a square SS 304 specimen by the µ-SED process. A scanning electron microscopic image of a mesohole fabricated by the SED process using a rotating hollow brass electrode of 3 mm diameter is shown in Figure 8.

### 1.4. Process Parameters of µ-SED and Their Effect on the Responses

**Figure 9:**
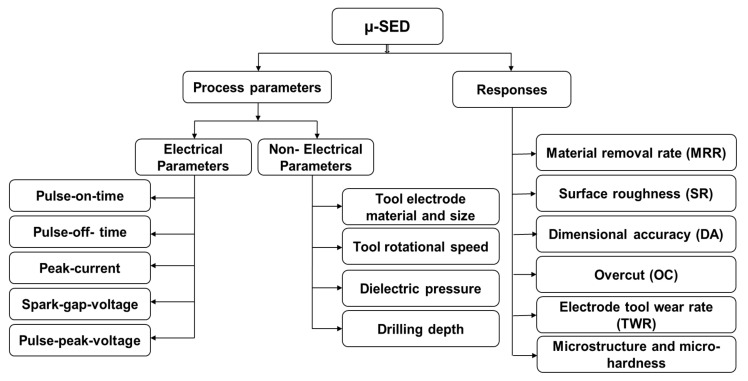
Micro spark-erosion drilling (µ-SED) process parameters and responses.

Figure 9 shows the process parameters and responses considered in the µ-SED process. The process parameters of µ-SED and µ-SEM are similar except the rotation of the tool electrode. The process parameters of µ-SED can be classified into two categories namely (i) electrical parameters; and (ii) non-electrical parameters. Electrical parameters are pulse-on-time, pulse-off-time, peak current, spark-gap voltage, pulse peak voltage, and capacitance; while tool rotational speed (TRS) of tool electrode, tool electrode feed, tool electrode diameter, drilling depth, workpiece material and thickness, and dielectric pressure/flow rate are non-electrical parameters. These parameters can be varied during drilling fine holes. The electrode materials, dielectric fluids, workpiece thickness, and aspect ratio are other important parameters but cannot be varied during the drilling operation. The higher values of pulse-on-time, peak current, pulse peak voltage, tool rotational speed, and tool electrode feed increase the material removal rate (MRR), electrode tool wear rate (TWR), and surface roughness (SR) of microholes. The higher values of pulse-off-time decrease the MRR, TWR, and SR of microholes. Tool electrode vibration due to loose clamping in the tool holder and loose clamping of the workpiece are responsible for the overcut and poor dimensional accuracy of microholes fabricated by µ-SED. Proper flushing is necessary for smooth drilling to achieve accurate and precise microholes. A higher dielectric flow rate is desirable to flush away the eroded particles from the drilling zone and avoid their redisposition on the drilled hole and the tool electrode surface. Redeposition of eroded particles is responsible for the formation of the recast layer and short circuiting [21,22,23]. Tool and workpiece materials also affect the MRR. Higher MRR can be achieved using workpiece and tool materials that are more electrically conductive. The high rotational speed is recommended for higher MRR and surface finish. µ-SED is more accurate and faster than µ-SEM process.

### 1.5. Applications and Advantages of the Micro Spark-Erosion Drilling Process

µ-SED is mostly used for quick, accurate, and fine deep-hole drilling applications. µ-SED can be found in several industrial applications, such as dental and medical, automobile, dies and moulds, electronics and telecommunication, and aerospace industries. The products and components which consist of microholes drilled by µ-SED are: (i) spray/injection nozzles; (ii) metal filters; (iii) fuel injectors; (iv) metallic filters; (v) dental tools; (vi) coolant holes; (vii) cooling manifolds; (viii) oil drain; and (ix) turbine blades.

The major advantages of µ-SED are (i) the ability to drill deep high-aspect-ratio (HAR) fine holes, (ii) the ability to produce accurate fine circular holes, (iii) no burr formation, thus eliminating the secondary finishing process, (iv) ability to drill high precision microscopic-holes, (v) the absence of mechanical stresses, (vi) the ability to produce holes on any kind of materials irrespective of their hardness, (vii) the ability to produce holes on thin and fragile materials; (viii) the ability to drill holes at a certain angle, (ix) the high material removal rate; and (x) unattended machining for a longer period.

Apart from the abovementioned advantages, µ-SED has certain limitations, such as the excessive tool electrode wear, not being suitable for non-circular holes, not being suitable for large-sized holes, the debris accumulated between IEG, and the abnormal sparks during drilling.

In the past two decades, substantial research has been focused on drilling deep fine holes in a variety of materials using various conventional and advanced micro-drilling methods. Nevertheless, very limited research work is found in the literature on the drilling of deep fine holes on difficult-to-machine materials (i.e., superalloys, smart materials, and shape memory alloys) using conventional methods. The following sections present a summary of the previous work on the fabrication of microholes by spark-erosion-based processes and their comprehensive discussion.

## 2. Review of Previous Research Work on Drilling Deep Fine Holes by Spark Erosion Machining (SEM) Processes

The available literature shows the interest of several researchers in the past few decades in exploring these drilling methods due to their exceptional abilities for net-shape drilling of fine holes on difficult-to-machine materials with better geometrical and dimensional accuracy, minimum tool electrode wear, and improved productivity. Table A1 summarizes the previous work done on the drilling microholes by spark erosion machining (SEM) and spark-erosion drilling (SED) and their micro version. The following sections present the critical evaluation of the previous work done on the drilling of deep fine holes using SEM, SED, µ-SEM, and µ-SED.

Lee et al. [24] successfully fabricated through small-sized microholes of 1.54 mm and 2.57 mm diameter on 6.2 mm thick SKD11 tool steel by SEM using a copper–tungsten electrode of 1.5 mm diameter. A novel measurement method for sensing electrode parameters in SEM drilling using a machine vision system was developed by Huang [25]. SKD11 steel was selected as a workpiece and copper electrode (with varying diameters of 2.0, 2.5, and 3.0 mm) as tool materials. Eight experiments were carried out by varying the peak current, tool electrode diameter, and machining depth. They reported an increment in the drilling speed with the peak current but that compromises with tool wear. In an interesting study, Jahan et al. [26] proposed an analytical model to estimate the efficacy of workpiece vibration at a lower frequency during machining deep microholes by µ-SEM. The model was validated by experimental results. They fabricated through microholes on WC of Grade MG18 by µ-SEM using a pure tungsten electrode of 200 µm diameter. A group of researchers conducted a total of sixty experiments with different drilling parametric combinations to validate the proposed model to understand the effect of the high-aspect-ratio microholes fabricated on stainless steel 304 sheets by horizontal and vertical µ-SEM using different diameters of tungsten electrodes [27]. They reported that (i) the experimental results are close to the theoretical model values; (ii) the differences between the theoretical and the real values due to debris, temperature, and electrode rotation in the discharge gap. The high-aspect-ratio through microholes of 300 µm diameter were fabricated by a µ-SEM process using hydrocarbon oil (HEDMA 111) as the dielectric to identify the effect of different electrodes namely copper, brass, tungsten carbide (cylindrical and tubular: OD: 300; ID: 120 µm), and 1 mm thick workpiece materials, namely stainless steel (AISI 304), titanium (Grade 2), magnesium (AZ31B-F), and brass (CuZn35) on the tool wear ratio [28]. It was reported that the tungsten–carbide electrode has a minimum tool wear as compared to other selected electrodes. Selvarajan et al. [29] successfully fabricated microholes on MoSi2–SiC composites by µ-SEM using cylindrical copper tube electrodes. After a comprehensive investigation, they reported that (i) current and pulse-on-time significantly affect the multiple performance characteristics in the SEM drilling; (ii) they obtained higher MRR with better accuracy microholes by increasing current, pulse-on-time, spark gap, and dielectric flow rate, and decreasing pulse-off-time; and (iii) MRR, TWR and selected geometrical tolerances were improved together by using the proposed optimization method. An effective model was developed in an important investigation to estimate the electrode wear during SEM drilling [30]. A copper electrode with the inside diameter of Ø 0.7 (Ø 0.25 mm) was used to fabricate through mesoholes on the SKD-11 workpiece by SEM process. A total of ten experiments were conducted using different parametric combinations to identify their effect on tool wear ratio. The developed model estimates the accurate prediction of the tool electrode wear ratio. The prediction error is less than 3%.

Zhang et al. [31] fabricated high-aspect-ratio through microholes on 200 µm thick stainless steel by µ-SEM using tungsten electrodes in the presence of oil served as dielectric during drilling. They found less than 2 µm repeated drilling accuracy of micro-electrodes during drilling microholes by µ-SEM. They also achieved ±1.1 µm accuracy of array micro holes. Li et al. [32] conducted a comparative study on three different shape electrodes of tungsten–carbide to fabricate a high-aspect-ratio microhole of 300 µm diameter on 0.91 mm thick SUS 304 by µ-SEM. The tool electrodes of the designed diameter for conducting experiments (i.e., five experiments) were prepared by wire-assisted spark-erosion-machining (WSEM) process. They reported that a short pulse is suitable for fabricating microholes by µ-SEM. 

Nine trial experiments were designed and performed using a full-factorial design (23) to identify the significant drilling parameters for SEM and laser drilling [33]. The trial experiments aimed to obtain the optimized parameters for the main experiments. SEM and laser drilling were used to fabricate microholes of 800 µm diameter on 5 and 10 mm thick nickel-based aerospace alloy (Inconel 718 nickel-based super-alloy) at high speed. They reported that (i) SEM is the most suitable for microholes in terms of accuracy and precision, while laser drilling is superior in terms of drilling speed; (ii) SEM recast layer thickness of 10–15 μm as compared to ~80 μm by laser drilling; and (iii) laser drilling time is less than 3 s as compared to 48 s by SEM for considered 10 mm thick workpiece. D’Urso et al. [34] proposed an acquisition system for the measurement of the variable parameters of µ-SEM and statistically analyze their effect on the considered responses, namely drilling time and tool electrode wear. The experiments were conducted by fabricating through microholes of 300 and 150 µm diameter on 5 mm thick 316 L stainless steel by µ-SEM using copper tubular electrodes having outside diameters of 300 and 60 µm and inside diameters of 150 and 120 µm. The exchanged power was considered as an adjustable parameter to identify the influence of the peak current and voltage on the experimental results. The experiments were planned using the full factorial design of experiment technique by varying the peak current and a voltage (both variables have two-levels), and each experiment was repeated five times. They concluded that MRR increased with the increasing exchanged power using Φ 300 mm electrode. Opoz et al. [35] fabricated high-aspect-ratio through microholes of 400 µm diameter that were drilled on high speed steel I.3343/M2 by µ-SEM using a circular tungsten carbide rod and machining oil as the dielectric (i.e. composition of minerals and synthetic oil). The experiments were conducted to identify the effect of the electrode materials on MER, overcut and tool wear during drilling. They reported that (i) µ-SEM has potential to fabricate microholes; (ii) better geometrical accuarcy of microholes can be achieved by µ-SEM; and (iii) TWR increases with drilling feed.

Macro-holes of 1 and 2 mm diameter were fabricated on Titanium TC4 by SEM process using a copper electrode and deionized water as the dielectric [36]. Experiments are carried out to validate the 3D simulation model based on the gap flow field with flushing and self-adaptive distribution using a solid-liquid two-phase flow equation. The results of experimentation confirmed that the drilling mark at the bottom surface of the hole and the tapered wall is similar to the simulation results. To identify the influence of different dielectric fluids (water and boron carbide additives mixed with water) on machining time and tool electrode wear, some researchers conducted a comparative study [37]. They fabricated a high-aspect ratio through microholes of 215 µm diameter on 5 mm thick C17200 beryllium–copper by µ-SEM using cylindrical tungsten electrode of 200 µm diameter with a rotational speed of 2000 rpm and deionized water serving as a dielectric. Further, the surface finish of drilled microholes was improved by in-situ powder-mixed spark erosion machining (PMSEM) with boron carbide additives mixed with water dielectric. They concluded that (i) it achieved higher drilling speed and lower tool electrode wear using water as dielectric than kerosene; (ii) the values of electrode wear, average diameter, and taper angle increases with an increase in current; and (iii) the application of in-situ PMSEM with boron carbide additive mixed with water significantly improved the average surface roughness 2.65 µm to 0.92 µm. Okada et al. [38] fabricated through curved microholes on the alloy tool steel SKD11 and aluminium alloy A5052 by µ-SEM using ball electrode CuW (φ 5.5 mm) and oil as a dielectric. They studied the influence of discharge current, pulse duration, duty factor, servo voltage, and vibration amplitude on MRR. They concluded that (i) electrode shape error is less than 0.5 mm; and (ii) the ball electrode improved the drilling performance. An innovative computational fluid dynamics model (CFDM) was successfully proposed to estimate the capability of the vacuum-assisted debris flushing system and optimization of parameters in the fabrication of deep holes by µ-SEM [39]. Wang et al. [40] proposed the flushing method which is the combination of tube-inner and tube-outer dielectric under high pressure and high velocity, respectively, to improve the machining efficiency during machining microholes. The experiments were carried out by manufacturing microholes of 480 µm diameter on Inconel 738 by a µ-SEM process using hollow circular Brass H65. They reported that (i) they improved drilling efficiency by ~ 89%; in the breakthrough-hole stage; (ii) the accumulation of eroded particles can be reduced by ~94% using the proposed flushing method. Skrabalak et al. [41] investigated the influence of tool electrode length during drilling microholes by the µ-SEM process. Microholes of 0.11 mm diameter were fabricated on 1 mm thick 304 L stainless steel (10 samples) by µ-SEM using tungsten–carbide rod 0.1 (−0.01) mm and HEDMA oil as the dielectric. The outcomes of their work are (i) short length drills are most suitable for microholes; (ii) they achieved less machining time by a short drill; (iii) the machining cost is less for medium drill; and (iv) they observed negligible effect in TWR by various electrode lengths.

Bellotti et al. [42] fabricated through microholes of 290 µm diameter on 2 mm thick Ti-6Al-4V by µ-SEM using brass tubular electrodes (290 µm outside diameter and 120 µm inside diameter). They observed no conical taper angle at the bottom of microholes with the help of the developed method. Li et al. [43] fabricated through microholes on 6 mm thick Inconel 738 by µ-SEM and electrochemical machining (ECM) using a hollow brass electrode of 0.5 mm diameter. Lower values of voltage and current were found suitable for the removal of the recast layer using ECM.

A comparative study was conducted for drilling macroholes on die steel by SEM using nickel-coated and diamond–nickel-coated composite electrodes of 2 mm diameter with each using deionized water for flushing eroded particles during drilling [44]. They concluded that (i) length wear and side wear were significantly reduced by diamond–nickel-coated composite electrodes, and (ii) nickel coating improves the wear of tool electrodes. Liu and Bai [45] investigated the axial wear of tool electrodes during the machining of an array of microholes on stainless steel by a µ-SEM process using deionized water as a dielectric. With the increase in depth–diameter ratio, axial wear of electrodes was increased.

Sánchez et al. [46] developed a setup to hold the low diameter tool electrode for drilling microholes on difficult-to-machine materials by SEM-drilling. This setup can hold copper–tungsten (CuW) and graphite electrodes of varying diameters to fabricate microholes by SEM-drilling. They successfully manufactured a 4.15 mm deep microhole on Ti6Al4V using a 300 µm diameter CuW electrode. Yu et al. [47] developed a new approach for more effective self-flushing having planetary movement for fabricating high aspect ratio (18) through microholes and complex-shaped blind microholes by µ-SEM. This approach is also used for fabricating blind noncircular microholes. Fu et al. [48] used a 40 μm diameter of electrodes made of pure tungsten and a tungsten–carbide alloy to determine the effect of electrode materials on tool wear rate, and the side gap in the drilling of microholes by the µ-SEM process using machining oil (CASTY-LUBE EDS) and deionized water as the dielectric. They concluded that (i) TWR is more in machining oil as compared to deionized water; (ii) side gap-width increases with the increase in the drilling feed and drilling energy; (iii) tungsten electrodes produce more gap-width; (iv) TWR increases with machining feed; and (v) more TWR in tungsten–carbide electrodes than a bare tungsten electrode. Rashid et al. [49] optimized the SEM parameters for drilling deep microholes on mild steel (AS3679) by SEM process using a 1 mm diameter of hollow copper (Cu) electrode. Taguchi’s L9 orthogonal array was used to conduct nine drilling experiments by varying pulse-off-time, peak current, and servo voltage. They concluded that higher values of pulse-off-time, peak current, and medium values of servo voltage are suitable to achieve the better dimensional accuracy of microholes. Debnath and Patwari [50] fabricated arrays of square microholes by µ-SEM using a tool electrode having an array of nine (3 × 3) square micro-bars.

## 3. Review of Previous Research Work on Drilling Deep Fine Holes by Spark Erosion Drilling (SED) Process

Jeong et al. [51] proposed geometric-based simulation models of the SED process using a rotating cylindrical electrode as a tool to calculate the tool and through-hole geometry. Tool rotation, the spark gap, pulse duration, single spark crater, and tool wear rate (TWR) were considered as simulating parameters for accurate analysis of tool and through-hole geometry. Models were successfully validated with experimental results. Using an electrolytic copper tube (outside diameter 3 mm and inside diameter 0.8 mm) as tool electrode and commercial SEM oil, a high aspect ratio through small-sized holes on Inconel 718 by SED process were also fabricated [52]. Based on a response surface methodology integrated experimental study, it was reported that peak current, duty factor, and TRS significantly affected MRR, and peak current and pulse on-time affected surface roughness. The influence of the high rotational speed of a tool electrode (50,000 rpm) on MRR and the accuracy of microholes were studied during drilling of a high-aspect-ratio through a microhole of 125 µm diameter on 100 µm thick stainless steel (SUS304) by µ-SED using tungsten rod electrode (φ 125 µm). It was observed that many high-aspect-ratio microholes can be drilled with improved straightness and with minimum tool wear ratio by using higher tool rotational speed (TRS) [53].

Meso-sized holes were fabricated on tool steel (X153CrMoV12-1) by SED process using brass (CuZn37) electrodes of 2 mm diameter with spindle rotational speeds of 500 revolutions per minute and negative polarity [54]. A total of four experiments was conducted to identify the effect of dielectric flow rate (ranging between 5 l/h and 25 l/h) on drilling speed (DS), electrode tool wear rate (TWR), and surface roughness (SR). It was reported that (i) TWR and recast layer reduced with increasing dielectric flow rate; (ii) it achieved uniform wall surface of macro-sized holes with low dielectric flow rates; and (iii) surface roughness slightly increased with higher dielectric flow rate.

A comparative study was performed to evaluate the effect of dielectric (i.e., kerosene and deionized water) fluids on the quality of an array of microholes (256 holes) fabricated on stainless steel (17–7 PH) by µ-SED using a pure tungsten electrode of 100 µm diameter [55]. The flow field simulation was used to analyze the flow of deionizing water and validated by experiments. They concluded that (i) deionized water is more suitable for drilling arrays of microholes; and (ii) the maximum microholes diameter deviation is 1.3 µm [55]. Some blind microholes were fabricated on Monel K-500 by a µ-SED process using a tungsten electrode of 300 µm diameter [56]. A detailed experimental study based on the full factorial design was conducted and it was found that gap voltage, capacitance, and depth of microhole were significant factors for selected responses. Moreover, Ra in the range of 6.2 µm to 6.6 µm was obtained.

In an important study, Plaza et al. [57] proposed a helically shaped electrode to ensure easy removal of debris during the drilling of deep microholes. They fabricated high-aspect-ratio (10:1) through microholes on Ti6Al4V by µ-SED using a graphite electrode of 301 µm diameter and a CuW electrode of 313 µm diameter with a rotational speed of 40 rpm to identify the effects of electrode materials, open voltage, capacity, and drilling speed on MRR, TWR, drilling time, and quality of microholes. They achieved a 37% reduction in drilling times by the proposed helical electrode.

In a comprehensive investigation, fifty-four experiments were planned and conducted using Taguchi’s L54 mixed orthogonal array to fabricate through microholes of 0.5 mm diameter on 316 L stainless steel sheet using µ-SED using a tungsten rod with a diameter of 300 µm and DCO-1000i SEM oil serving as a dielectric [58]. Taguchi-based grey relational analysis (GRA) was employed to obtain the optimum drilling parametric combination. They concluded that a higher MRR and a lower value of TWR can be achieved when the tool is used as a negative polarity. They also observed unstable machining at zero rpm. Sapkal and Jagtap [59] fabricated through microholes of 500 µm diameter on 2.5 mm thick titanium alloy (Ti-6Al-4V) by the µ-SED process using 0.5 mm diameter of copper–tungsten (CuW) electrode and SEM dielectric oil. Based upon a detailed experimental study, they found that (i) MRR is influenced by TRS, capacitance, and discharge parameters; (ii) capacitance has the major contribution to the side gap; and (iii) they achieved higher MRR with an increase in TRS. Kumar et al. [60] fabricated through microholes with a diameter varying in the range of 0.3 to 3 mm on a Ti-6Al-7Nb workpiece by µ-SEM using the hollow electrode. They conducted preliminary experiments to select the ranges of µ-SED variable parameters for further experimentation. The effects of peak current, pulse-off time, pulse-on time, working material thickness, and drilling angle on considered responses, such as drilling rate and TWR, were determined during preliminary experiments. The results are (i) the fabricated high-aspect-ratio microholes by µ-SED; (ii) the observed taper in microholes with increasing hole depth; (iii) the DR and TWR increase with the increase in peak current and pulse-on-time; and (iv) the achieved stable drilling with higher DR and lower TWR at machining condition of 60 μs pulse-on-time; 50 μs; pulse-off-time; 4 A peak current. Taguchi’s based orthogonal array was used to plan and conduct sixteen experiments for fabrication of precision through microholes on 20 µm thick metallic glasses Zr60Cu30Ti10 by µ-SED using a tungsten electrode of 513 µm diameter [61]. They found that TRS has significant parameters responsible for the geometrical accuracy of drilled microholes and drilling time.

High-aspect-ratio through microholes on 2 mm thick Ti6Al4V alloy by µ-SED using brass tubes (outside diameter 350 µm and inside diameter 130 µm) with a 750 rpm rotational speed and hydrocarbon oil as the dielectric were fabricated [62]. Another important study reports the fabrication through microholes of 500 µm on a 10 mm thick Inconel 718 rectangular plate by the µ-SED process using a brass tubular electrode of 0.5 mm diameter with a rotational speed of 135 rpm and demineralized water as the dielectric [63]. The peak current produced a significant effect on MRR while pulse-on-time on the taper angle of the microholes.

Kuppan et al. [64] studied the effect of SED parameters on surface roughness, overcut, and hole profile of deep microholes fabricated on Inconel 718. Electrolytic copper electrodes were used to fabricate 62 mm deep through holes of varying diameters (ranging from 3.055 to 3.316 mm) on Inconel 718 with a thickness of 62 mm by SED process. They observed that (i) peak current and pulse on-time are a significant influence on surface roughness; (ii) pulse-on-time is a significant parameter for radial overcut; (iii) the best microhole profile can be achieved at 4 A peak current, 40 µs pulse on-time, and 300 rpm TRS; and (iv) sticking eroded particles are observed at the top and the bottom of sidewalls. Xu et al. [65] fabricated stepped micro-holes on YG8 cemented carbide by µ-SED using a micro milling cutter as a tool electrode of 400 μm diameter. They concluded that the best quality of stepped micro-holes can be achieved at 2000 RPM rotational speed, 90 V drilling voltage, and 0.5 duty ratio.

## 4. Conclusions and Future Research Directions

The spark-erosion-based drilling of fine holes and a review of the past work on that have been reported in this paper. The conclusion is as follows:

- µ-SED can produce high-quality circular microholes on difficult-to-machine materials.
- µ-SEM has the potential to produce different types of microholes.
- Tool rotational speed is a significant parameter to improve the productivity of µ-SED.
- Most of the work has been focused on fabricating circular microholes by µ-SED.
- Most of the work focused on TWR during the drilling of microholes by µ-SEM and µ-SED.
- Very little work has been done on the modeling and optimization of µ-SEM and µ-SED variable parameters for drilling microholes.
- µ-SED is more accurate and faster than the µ-SEM process.

The following aspects can be explored as future work to further establish the field of meso and microhole drilling by spark erosion based machining processes:

- Investigations on fabricating microholes by micro-drilling and other advanced machining processes.
- Investigations on the fabrication of tapered microholes by µ-SED process.
- Investigations on the sustainability of µ-SED process for fabrication of microholes.
- Identify the effect of different dielectrics and wire materials on the performance of µ-SED process for the fabrication of microholes.
- Development of hybrid drilling processes, such as µ-SED and ECH processes.
- Analysis of microholes quality, namely geometrical accuracy, surface finish, and surface integrity.
- Exploring other advanced drilling processes for the fabrication of microholes.
- Improving the productivity of µ-SED for drilling microholes by optimizing the process parameters.
- Exploring µ-SEM for drilling conical microholes.
- Comparative evaluation among µ-SED, µ-SEM, advanced machining processes, and non-traditional drilling processes for fabricating microholes.

## Figures and Tables

**Table 1:** Commonly used drilling methods for manufacturing miniature holes.

Type	Methods	Nature of the Process	Manufacturing Process
Conventional drilling methods	Chip formation	Subtractive	Micro-lathe, micro-milling, micro-drilling
Advanced drilling methods or Non-conventional drilling methods	Mechanical erosion drilling	Erosion	Abrasive water jet drilling (AWJD) and Ultrasonic drilling (USD)
	Thermoelectric erosion drilling	Controlled spark erosion	SEM, SED, and their micro-version (µ-SEM and µ-SED)
		Thermoelectric vaporization	Laser drilling (LD)
	Chemical drilling	Chemical ablation	Chemical drilling (CHD)
	Electrochemical drilling	Ion displacement	Electrochemical drilling (ECD)

## Data Availability

The study did not report any data.

## Appendix A

**Table A1:** Summary of the previous research on fabrication of high precision deep fine holes by SEM, µ-SEM, SED, and µ-SED processes.

Researchers (Year) [Ref. No.]	Microhole Specifications	Workpiece, Tool Electrode, and Dielectric	Process Parameters	Responses	Key Findings/Remarks
Huang (2008) [25]	Mesohole (Through hole) 1.5-3.0 mm	SKD11 tool steel Copper electrode	Peak current; tool electrode diameter; and machining depth	Tool electrode wear; and drilling speed	Developed a novel measurement technique for detecting rotary tool parameters in SEM drilling using a machine vision system. Drilling speed and TWR increased with an increase in peak current.
Jahan et al. (2012) [26]	Microhole (Through hole) (Dia.: 200 µm) Depth: (1, 1.5, 2 mm)	WC of Grade MG18 (Thickness: 1, 1.5, 2 mm) Pure tungsten (OD: 200 µm)	Workpiece vibration; gap voltages; and capacitances	Drilling performance; surface quality; and dimensional accuracy	Suggested a diagnostic model estimate the efficacy of workpiece vibration at a lower frequency. Observed short frequency vibration more effective at the small discharge energy level.
**Researchers** **(Year) [Ref. no.]**	**Microhole** **specifications**	**Workpiece, tool electrode, and dielectric**	**Process parameters**	**Responses**	**Key findings/Remarks**
Li et al. (2013) [27]	Microhole Diameter: 156.94 µm Depth:	Stainless steel 304 sheet (Thickness: 500 µm) Tungsten electrode Kerosene oil	Open circuit voltage; electrode diameter; capacitance; and dielectric	Drilling time, electrode feed; and TWR	Proposed a theoretical model-based to identify the effect of the high aspect ratio of a microhole drilled by µ-SEM. The suggested model was successfully confirmed with experimental outcomes.
Selvarajan et al. (2015) [29]	Mesohole, (Through hole) Dia.: - µm Depth: 4 mm	MoSi2–SiC composites Copper tube electrode	Current; pulse-on-time; pulse-off-time; spark-gap; and dielectric flow rate;	MRR; TWR; circularity; cylindricity; and perpendicularity	Developed a novel methodology for the optimization of SEM. drill parameters. Performed multi-objective optimization. Achieved higher MRR with better accuracy of microholes.
Zhang et al. (2015) [31]	Microhole (Through hole)	Stainless steel (Thickness: 200 µm) Tungsten electrode Dielectric: Oil	Open voltage; pulse duration; and pulse interval	Dimensional accuracy	Observed less than 2 µm repeated drilling accuracy of micro-electrodes. Achieved ±1.1 µm accuracy of array micro holes.
Li et al. (2016) [32]	Microhole (Through hole); High-aspect-ratio Dia.: 300 µm, Depth: 0.91 mm	SUS 304 Tungsten carbide electrode (Dia.:250 µm) HEDMA oil	Frequency; energy; gap voltage; and current; voltage;	MRR; TWR; and surface quality	Identified short pulse most appropriate for microholes drilling by µ-SEM.
Antar et al. (2016) [33]	Microhole (Through hole) (Dia.: 0.8mm) Depth: 10 mm	Inconel 718 (5-10 mm thick) Tungsten Carbide electrodes (Dia.: 0.8mm)	**Laser:** Peak Power; Laser Speed; Pulsing Frequency; **SEM:** Current; compression; SBOX	Drilling speed; recast layer thickness; and hole taper	Achieved better geometric accuracy and precision by SEM. Secured higher drilling speed by the laser drilling process. Minimized recast layer thickness 10-15μm as compared to ~80μm obtained using laser drilling.
Zhang et al. (2017) [36]	Mesohole (blind and through hole) (Dia. 1and 2 mm)	Titanium TC4Copper electrode Deionized water	Flushing pressure; drilling depth; diameter; and tool velocity	Gap flow field; debris distribution; machining efficiency; and accuracy	Developed a mathematical model on the gap flow field with flushing and self-adaptive distribution using solid-liquid two-phase flow equation.
**Researchers** **(Year) [Ref. no.]**	**Microhole** **specifications**	**Workpiece, tool electrode, and dielectric**	**Process parameters**	**Responses**	**Key findings/Remarks**
Okada et al. (2017) [38]	Microhole (Through hole)	Alloy tool steel SKD11; Aluminum alloy A5052 Ball electrode CuW (Sф 5.5mm), Dielectric: oil	Discharge conditions; and vibration amplitude	MRR	Successfully drilled different types of curved microholes Observed electrode shape error less than 0.5 mm. Ball electrode improved the drilling performance
Wang et al. (2018) [40]	Microhole Height: 6 mm	Inconel 738 Hollow circular Brass H65; Length: 600 mm (OD: 480 µm ID: 240 µm)	Flushing pressure	Drilling efficiency	Proposed combined flushing method Drilling productivity considerably improved by ~ 89% in the breakthrough-hole step. Eroded particle accumulation can be minimized by ~ 94%.
Skrabalak (2018) [41]	Microhole (Through hole) (Dia.: 0.11 mm) Depth: 1 mm	304 L stainless steel (Thickness: 1mm) Tungsten carbide rod (OD: 0.1 mm) Dielectric: HEDMA oil.	Pulse width; pulse peak current; pulse frequency; voltage; and gap %	TWR; MRR; drilling efficiency; and drilling accuracy	Recommended short drill for microholes. Achieved less machining time by a short drill. Less drilling cost for medium drill. Observed negligible effect in TWR by various electrode lengths.
Bellotti et al. (2018) [42]	Microhole (Through hole) (Dia.: 290 µm) Depth: 2 mm	Ti-6Al-4V (2 mm thick) Brass tubular electrodes (OD: 290 µm; ID: 120 µm) Dielectric:HEDMA111	Width; frequency; current; open voltage; gain factor; and reference discharge voltage	TWR; MRR; and SR	Developed and tested a method based on the identification of anomalies in the short-circuit activities. Observed no conical taper at the bottom of microholes.
Liu and Bai (2020) [45]	Microhole (Through hole) (Dia: 80,100, 120 μm ) Depth: 100, 200, 300 μm	Stainless steel Deionized water	Applied voltage; and power mode	TWR	Axial wear of electrode increases with the increase in depth-diameter ratios. Observed uniform tool electrode wear.
Kuppan et al. (2008) [52]	Mesohole (Through hole) Depth: (25 mm)	Inconel 718 Electrolytic copper tube (OD: 3 mm; ID: 0.8 mm) Commercial SEM oil	Peak current; pulse on-time; duty factor; and TRS	MRR; average depth and SR	Peak current, duty factor, and TRS have a significant effects on MRR. Peak current and pulse-on-time have a major influence on Ra.
**Researchers** **(Year) [Ref. no.]**	**Microhole** **specifications**	**Workpiece, tool electrode, and dielectric**	**Process parameters**	**Responses**	**Key findings/Remarks**
Yahagi et al. (2012) [53]	Microhole (Through hole) High-aspect-ratio (Dia.: 125 µm; Depth: (100 µm)	Stainless steel (SUS304) (Thickness: 100 µm) Tungsten rod (ф 125 µm)	High tool rotation speed (TRS)	MRR and dimensional accuracy	Drilled high-aspect-ratio microhole with lower tool wear ratio at higher tool rotational speed (50000 rpm).
Munz et al. (2013) [54]	Mesohole (Through hole) OD: 2 mm;	Tool steel (X153CrMoV12-1) Brass (CuZn37) (ф: 2 mm)	Discharge current; pulse duration; duty factor; dielectric flow rate; and TRS	Recast layer; electrode wear; and SR	TWR and recast layer decrease with increased flow rate. Achieved uniform wall surface with low flow rates. SR slightly increased with a higher flow rate.
Zhu et al. (2014) [55]	Microhole Depth: 50 µm	Stainless steel (17-7PH); Pure tungsten (Dia.:100 µm) Kerosene and deionized water	Dielectric fluid, and TRS	MRR	Identified deionized water as more suitable for drilling an array of microholes. Observed 1.3µm as maximum diameter deviation.
Barman et al. (2014) [56]	Microhole (Blind hole) Depth: 6 mm	Monel K-500 Tungsten electrode of 300 µm diameter	Gap voltage; capacitance; and depth of hole;	Surface finish; and tool wear length	Observed gap-voltage, capacitance, and microhole depth as significant factors for selected responses Achieved Ra of 6.2 µm to 6.6 µm and Sa of 17 µm to 17.5 Observed 150 µm to 180 µm wear in electrode length.
Plaza et al. (2014) [57]	Microhole (Through holes) High-aspect-ratio (Dia.: 301 and 313 µm), Depth: (-)	Ti6Al4V Graphite (ф301 µm) and Cu-W (ф313 µm) electrodes; TRS: 40 rpm	Pulse-on-time; pulse-off-time; and gap-voltage	MRR, electrode wear, drilling time, and microhole quality	Successfully fabricated high aspect ratio microhole on Ti6Al4. Achieved 37% reduction in drilling times by helical electrode.
Pilligrin et al. (2017) [58]	Microhole (Through hole) (Dia.: 300 µm) Depth: 0.5 mm	316L stainless steel (Thickness: 0.5 mm) Tungsten rod (OD: 300 µm); Dielectric: DCO-1000i SEM oil	Tool speed and polarity; and tool speed	TWR; MRR; overcut; and taper angle	Achieved higher MRR and lower value of TWR using the tool as negative polarity. Observed unstable machining at zero rpm.
**Researchers** **(Year) [Ref. no.]**	**Microhole** **specifications**	**Workpiece, tool electrode, and dielectric**	**Process parameters**	**Responses**	**Key findings/Remarks**
Kuriakose et al. (2019) [61]	Microhole (Through hole) (Dia.: 513 µm) Depth: (20 µm)	Metallic glasses Zr60Cu30Ti10 (Thickness: 20 µm) Tungsten electrode (OD: 513 µm)	Capacitance; voltage; and TRS	Drilling time; taper angle; overcut; and geometrical deviation	Identified TRS as significant parameters responsible for better geometrical accuracy of microhole and minimum drilling time.
